# EVIDENCE OF PSYCHOSOCIAL BEHAVIORAL AND COGNITIVE FORTITUDE TO AD IN RACIALLY/ETHNICALLY MINORITIZED OLDER ADULTS

**DOI:** 10.1093/geroni/igae098.0620

**Published:** 2024-12-31

**Authors:** Alexandra Clark

**Affiliations:** The University of Texas Austin, Austin, Texas, United States

## Abstract

It is essential that emerging research focuses on characterizing important heterogeneity in risk and resiliency to Alzheimer’s disease (AD) within minoritized communities of color. Our previous work has used empirical methods to identify distinct psychosocial-behavioral and cognitive phenotypes within a large sample of community-dwelling Black and Latinx older adults enrolled in the Health and Aging Brain Study: Health Disparities (HABS:HD). This first part of this presentation will discuss the identification and characterization of unique psychosocial-behavioral phenotypes. We will highlight how these phenotypes displayed varied cognitive, neuroimaging, and AD biomarkers outcomes, with an emphasis on understanding mechanisms by which a “resilient” Low Resource/Low Distress group defies the odds of economic inequality and does not appear to be at increased risk for developing AD. This second part of this presentation will highlight data that provides a more nuanced understanding into why bilingualism has been linked to a later age of AD onset. We demonstrate that there is significant variability in the cognitive profiles of bilingual Latinx speakers that is partially attributable to language of testing, proficiency, dominance, and age of acquisition. Furthermore, we discuss the identification of a unique group of bilingual speakers with above average memory performance who are less likely to progress to the mild cognitive impairment phase. Collectively, this work illustrates the need to move beyond racial/ethnic group comparison studies to non-Latinx whites, highlights the importance of characterizing within-group heterogeneity, and further emphasizes potential points of prevention and intervention for optimizing aging outcomes in racially/ethnically minoritized older adults.

